# Novel Technique for Innervated Abdominal Wall Vascularized Composite Allotransplantation: A Separation of Components Approach

**Published:** 2014-09-19

**Authors:** Devinder P. Singh, Vasilios D. Mavrophilipos, Jeffrey A. Zapora, Jens Berli, Justin Broyles, Karan Chopra, Jennifer Sabino, Jamil Matthews, E. Bryan Buckingham, John S. Maddox, Rachel Bluebond-Langner, Ronald P. Silverman

**Affiliations:** Division of Plastic Surgery, University of Maryland School of Medicine, Baltimore, Md

**Keywords:** abdominal wall transplantation, component separation, chimerism, hernia, vascularized composite allotransplantation

## Abstract

**Objective:** Applications for Abdominal Wall Vascularized Composite Allotransplantation may expand if a functional graft with decreased immunosuppressive requirements can be designed. We hypothesize that it is anatomically feasible to prepare a functional, innervated, and vascularized abdominal composite graft using a multilayered component separation technique. Including vascularized bone in the graft design may decrease the immunosuppressive requirements by inducing immunologic chimerism. **Methods:** Two cadaver torsos were used. Adipocutaneous flaps were elevated from the midaxillary lines, preserving deep inferior epigastric artery perforators. A 2-layered component separation through the external and internal oblique fasciae was carried out, exposing segmental intercostal thoracolumbar nerves. Superiorly directed muscle release over the subcostal margin provided for a 3-rib segment with attached rectus abdominis muscle. The remainder of the full-thickness allograft was harvested with its vasculature. Flap inset into the recipient cadaver abdomen, with osteosynthesis fixation between donor and recipient ribs, was achieved. **Results:** The harvested grafts had an average size of 845 ± 205 cm^2^ with a total procurement time of 110 minutes. On one cadaver, 4 thoracolumbar nerves were isolated bilaterally, while the other cadaver yielded 3 nerves. The nerves were transected with an average length of 5.7 ± 1.2 cm. The graft vasculature was transected with a length of 4.40 ± 0.10 cm. **Conclusion:** Using the principles of component separation technique, we demonstrated a novel approach to harvest and transfer a neurotized osteomyofasciocutaneous abdominal wall allotransplant as a multipedicled, single functional unit.

Over the past 2 decades, vascularized composite tissue allotransplantation has been rapidly evolving. Successful reports of upper extremity,^1^ face,^2^ abdominal wall,^3^^-^6 trachea,^7^ larynx,^8^ and recently lower extremities^9^^-^10 indicate that vascularized composite tissue allotransplants (VCA) are both feasible and efficacious. The Abdominal Wall Vascularized Composite Allotransplant (AW-VCA) differs from the other VCAs in that it is currently only performed in conjunction with intestinal and/or multivisceral transplantation. In addition, it is the only nonfunctional composite tissue transplant routinely performed.

Since the first description by Levi et al a decade ago, 2 groups (University of Miami and University of Bologna) have published their consecutive case series of abdominal wall transplantation. In total, they have published the results of 18 denervated AW-VCA in 17 patients.^3^^-^6 Despite overall good graft and patient survival, this intriguing technique does not seem to have gained widespread acceptance. We believe this is due to the paucity of intestinal transplantations performed (n = 129 in 2011 according to the Scientific Registry of Transplant Recipients database) and the lack of a broader indication for AW-VCA.

We postulate the patient population that might benefit most from increased indications for abdominal wall transplantation without viscera or organ would be those people with massive “end-stage” hernias. Hernia disease burden in the United States is now epidemic and multifactorial in etiology. Patients with “end-stage” hernia disease, having total loss of domain with impaired biophysical and psychosocial function, represent a population with few viable options for the repair of the hernia and reconstruction of the abdominal wall. Because abdominal wall reconstruction is morbid, frequent, and often multirecurrent at initial presentation, there may finally be good reason and indication—in select patients—to consider a functional AW-VCA with immunosuppression for the reconstruction of massive composite defects.

Expanding the indications for AW-VCA evokes the ethical questions of subjecting a patient to a lifetime of immunosuppression. To justify such a risk, the AW-VCA procedure must first improve the biomechanical function of the graft and also minimize the immunosuppressive needs. Abdominal wall surgeons have learned that bridged mesh repairs are less durable than primary closure with functional reconstruction of the anterior fascia. Primary closure is most commonly achieved through component separation technique (ie, external oblique layer fasciotomy with subsequent rectus abdominis muscle flap medial advancement). We note the rectus abdominis muscle flap is not simply vascularized but, more importantly, also remains innervated when returned to its midline position of function with physiologic tension. In the same sense, it stands to reason that any AW-VCA graft should also remain innervated after transplantation to improve its function. As studies look to improve the practicality of nerve transfers in other VCA models, the same must be continued for AW-VCA.^11^

The minimization of immunosuppression and induction of immune tolerance reported in VCA has made large strides in recent years. Directly transplanting hematopoietic stem cells has shown to decrease the immunosuppressive burden due to its chimeric effects on the recipient.^12^^-^13 Studies at our institution have shown that simply including vascularized bone marrow with a VCA also has a chimeric effect that may decrease the immunosuppression requirements otherwise needed.^14^ The presence of vascularized bone marrow has already shown promising results with single agent maintenance therapy in upper extremity reconstructions.^15^ This concept of marrow-derived chimerism and future immunosuppressive regimens could help expand the indication for all vascularized composite allotransplants including the idea of abdominal wall allotransplantation without simultaneous solid organ or multivisceral transplant.

With the end-stage hernia patient in mind, drawing upon our large experience with component separation techniques, and improvement in VCA transplant biology, we now hypothesize that procurement of an innervated abdominal wall is technically feasible for allotransplantation. Our approach combines the concepts of functional hernia repair with the notion of innervated abdominal wall allotransplantation through preparation of the graft using a stepwise “multi-layered” component separation technique that carefully identifies individual segmental intercostal nerves beneath the internal oblique muscle. Secondarily, we hypothesize that an allograft can be designed with an osseous component to utilize the aforementioned immune-modulating chimerism effect.

## METHODS

**Figure 1:**
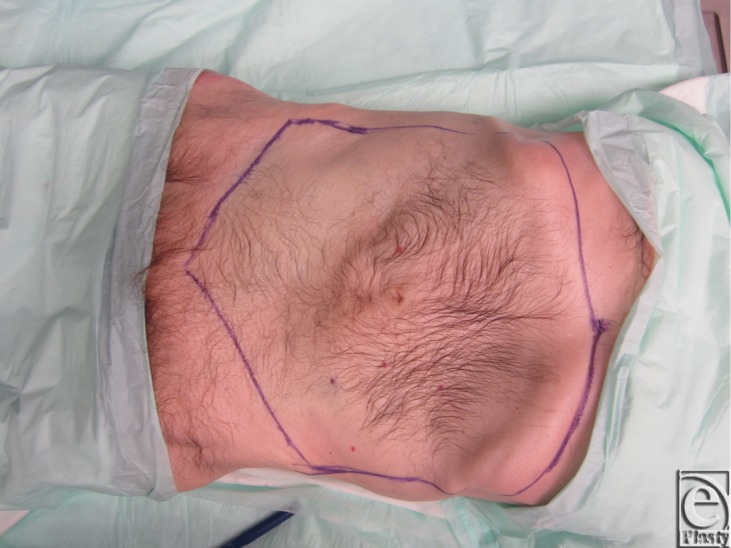
Hexagonal AW-VCA incisional outline indicated by blue markings.

**Figure 2:**
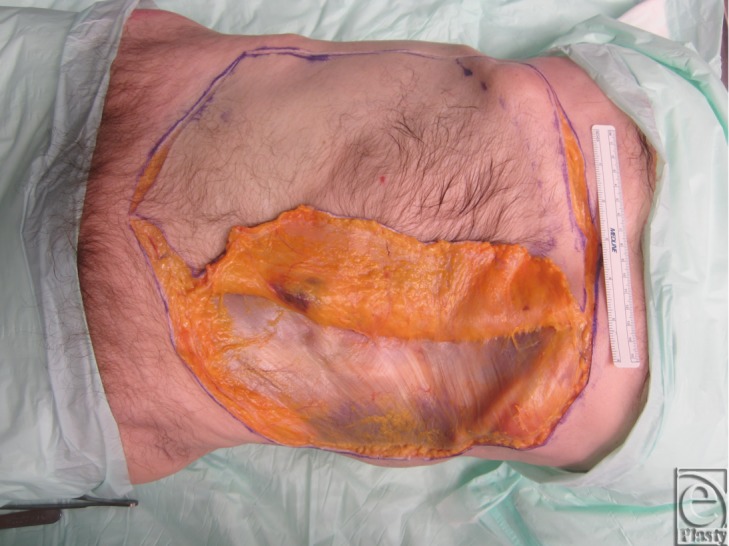
Adipocutaneous skin flaps elevated in a lateral to medial direction.

**Figure 3:**
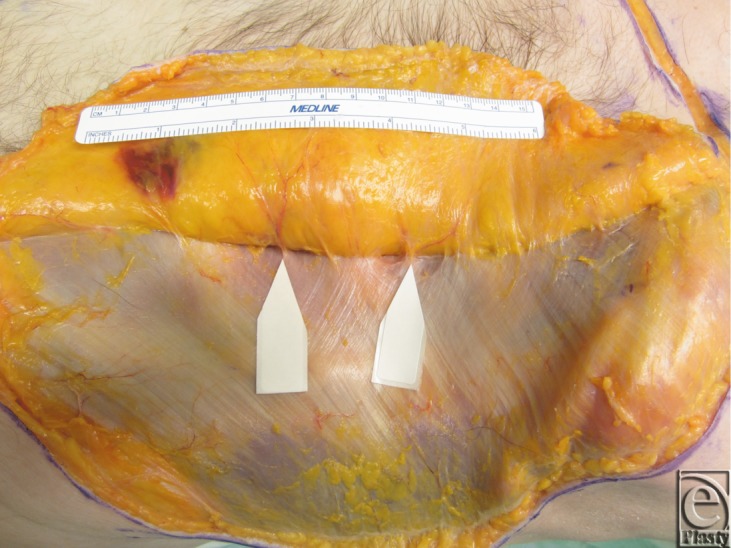
Deep inferior epigastric perforators (Huger Zone I) are preserved during skin flap elevation.

**Figure 4:**
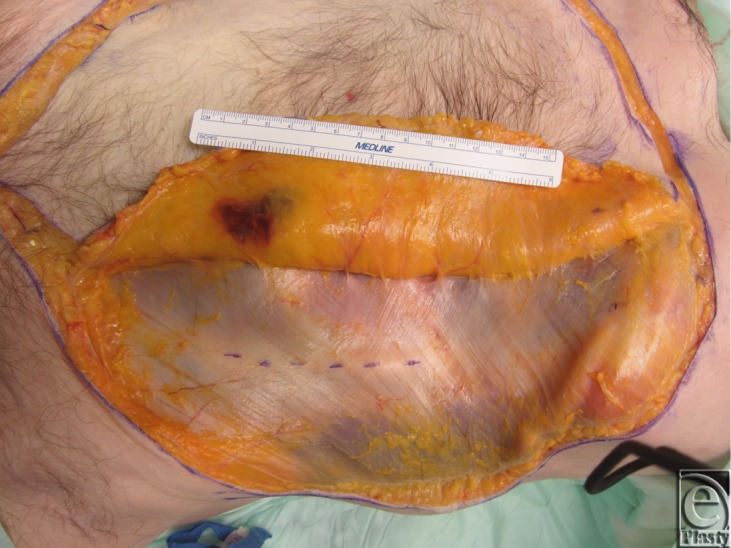
External oblique fascia with semilunar line indicated by dotted line. This is the site of the first layer component separation.

**Figure 5:**
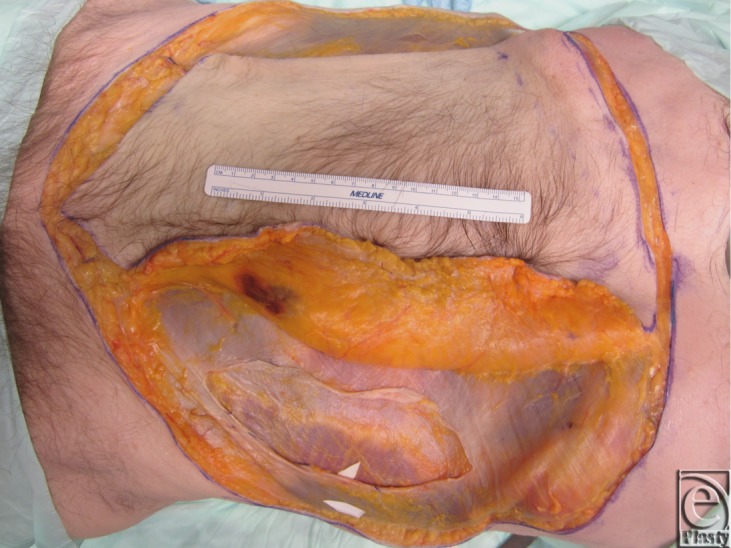
First layer component separation completed exposing internal oblique layer.

**Figure 6:**
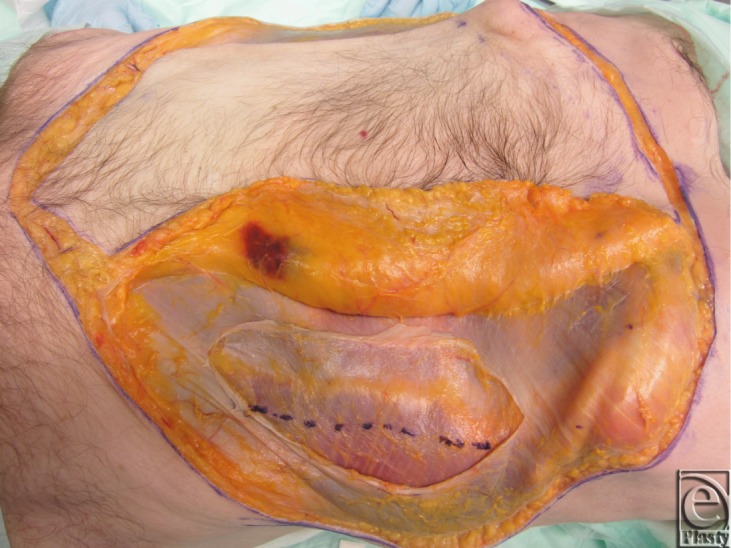
Demarcation of second stage of component separation 2 cm lateral to semilunar line.

**Figure 7:**
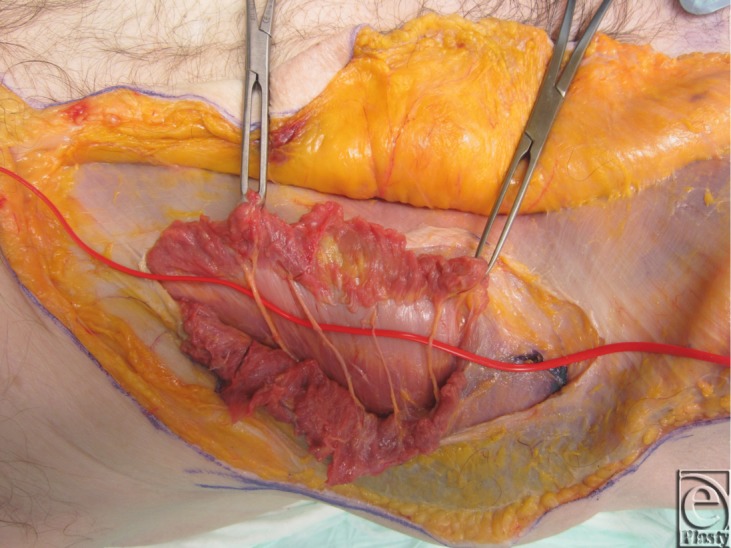
Segmental intercostal thoracolumbar nerves identified between the internal oblique and transversus abdominis muscle layer.

**Figure 8:**
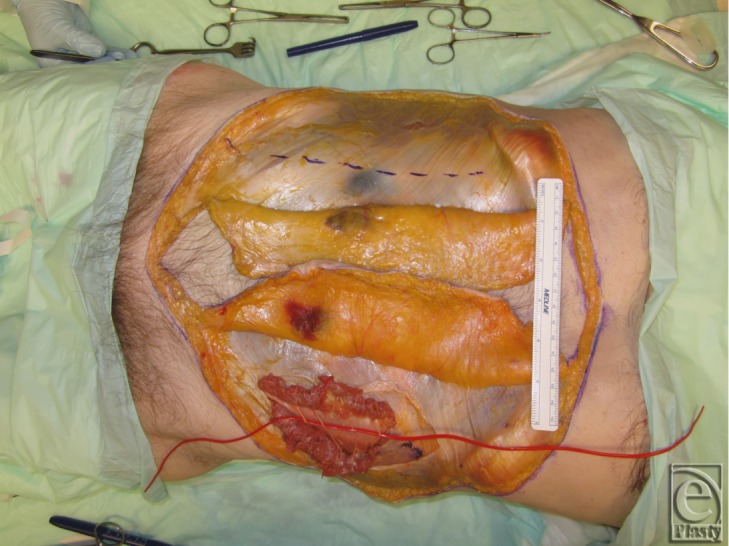
Left sided dissection complete, and skin flap elevation on the right.

Two fresh cadaver torsos were latex injected and obtained from the Maryland State Anatomy Board on June 6, 2013. The operative plan was for the procurement of an osteomyofasiocutaneous abdominal wall allotransplant, which could be neurotized and inset as a single multipedicled functional graft. To this effect, a hexagonal skin incisional was planned (see Fig 1). Incisions were carried down to the level of the abdominal fascia. Adipocutaneous skin flaps were elevated from lateral to medial direction on both sides of the torso, taking great care to preserve Huger Zone I perforating blood vessels from the deep inferior epigastric artery and vein (see Figs 2 and 3). The external oblique fascia lateral to the semilunar line was properly identified (see dotted line on Fig 4). Fasciotomy was carried along a vertical direction, exposing the internal oblique layer (see upperly directed arrow Fig 5). Internal oblique layer was then marked (see dotted line Fig 6) and carefully released as second stage of a “multilayered” component separation. Segmental intercostal thoracolumbar nerves were identified and dissected in continuity within the plane between internal oblique and transversus abdominis layers (Fig 7). These steps were repeated bilaterally (Fig 8).

**Figure 9:**
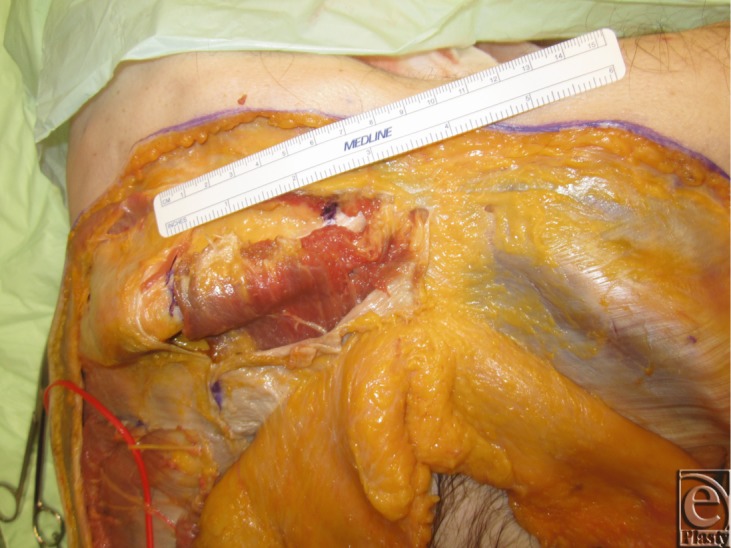
Rectus abdominis muscle attachment to lowermost ribs.

**Figure 10:**
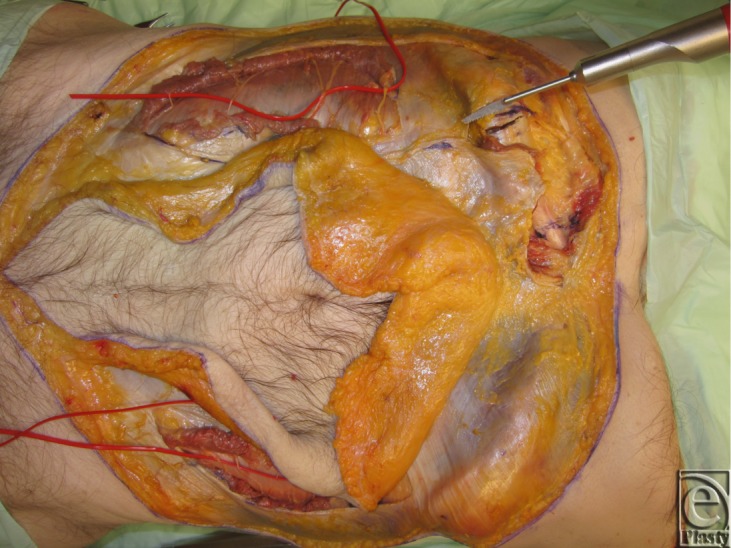
Release of 3 vertebrochondral ribs with attached rectus abdominis muscle.

**Figure 11:**
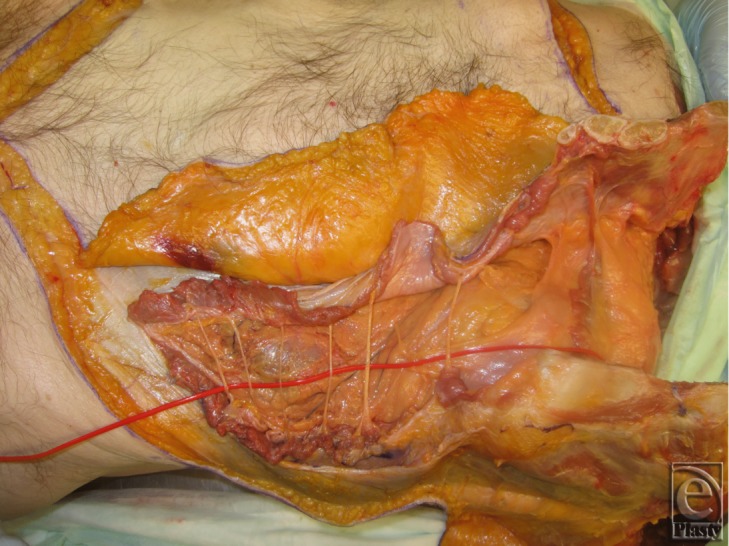
Complete release of vertebrochondral ribs with observable marrow.

Superiorly directed muscle release was carried up and over the subcostal margin, similar to the component separation modification used in the repair of subcostal or epigastric hernias; this maneuver revealed the attachments of rectus abdominis to the lowermost ribs (see Fig 9). Using a Stryker reciprocating saw and osteotome (see Fig 10), a 6-cm chest wall segment with 3 vertebrochondral ribs and attached rectus abdominis muscle was freed (see Fig 11). Right-sided internal mammary vessels were identified and preserved on cadaver 2.

**Figure 12:**
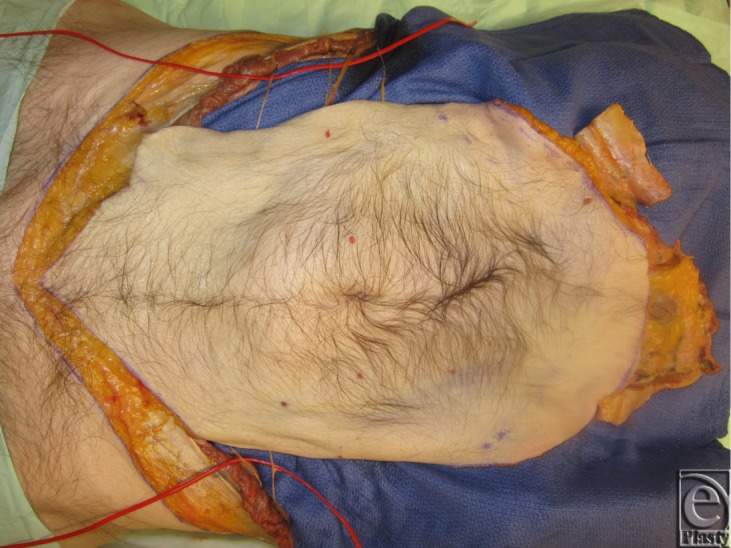
Full-thickness dissection of graft continued through the peritoneal cavity.

**Figure 13:**
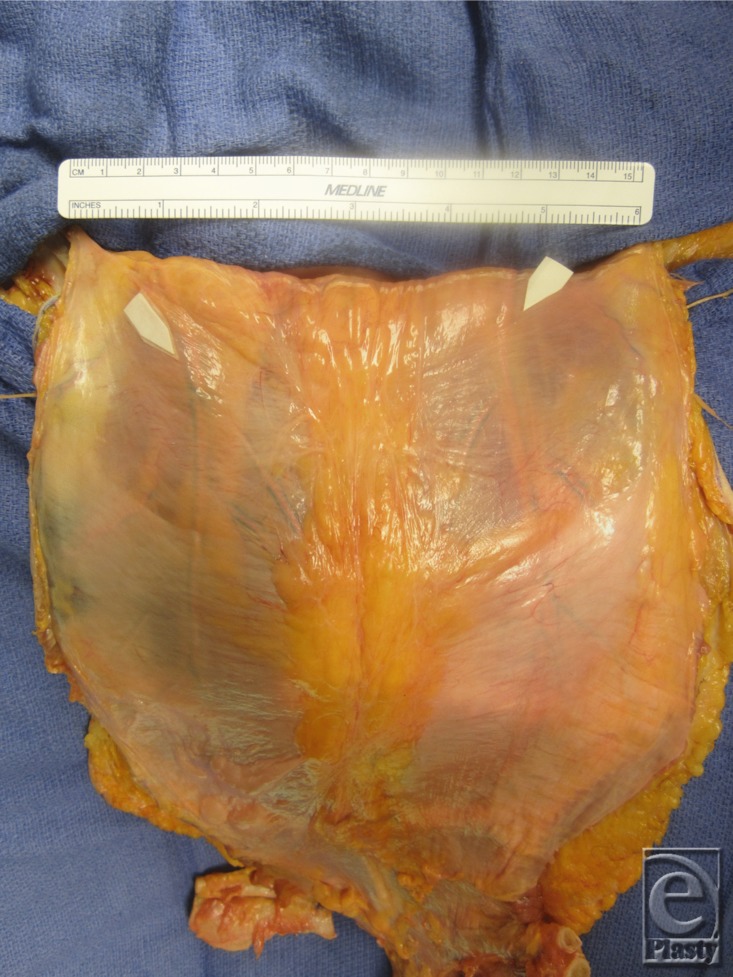
Graft was reflected in a cranial to caudal manner to identify and preserve the deep inferior epigastric pedicle indicated by the arrows. These pedicles provide the main blood supply to the graft.

**Figure 14:**
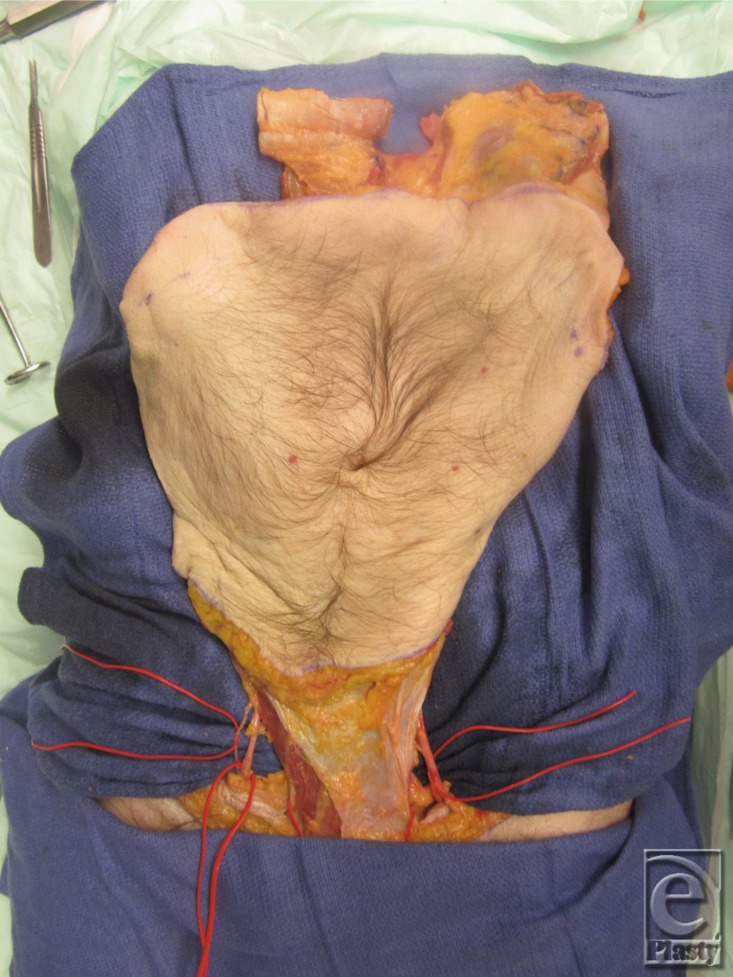
Dissection of the deep inferior epigastric pedicles.

**Figure 15:**
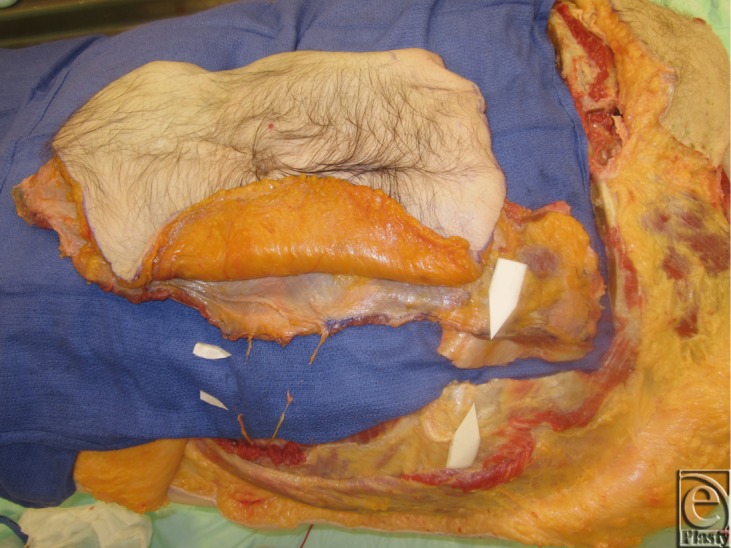
Graft transferred to recipient cadaver illustrating the potential nerve coaptation and bone synthesis.

**Figure 16:**
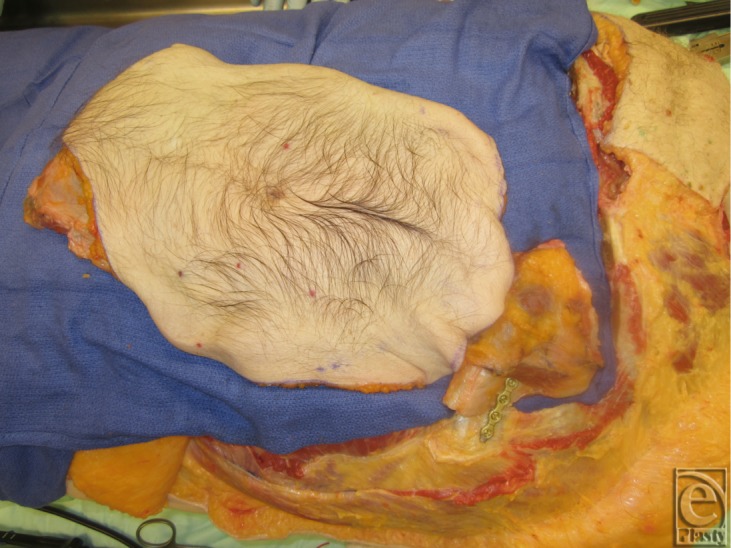
Simulated osteosynthesis between donor and recipient ribs using fixation plate and screws.

The allograft harvest was continued by carrying the dissection through to the peritoneum cavity beginning at the costal margin with full-thickness incisions in the abdominal wall between bony landmarks such as anterior superior iliac spine and symphysis (see Fig 12) and then reflecting in a cranial to caudal exposure (see Fig 13). The falciform ligament was encountered, ligated, and transected in this technique. The deep inferior epigrastric pedicle was then easily identified bilaterally (see Fig 13 arrows) running along the posterior rectus sheath, and it was fully dissected (see Fig 14) and transected bilaterally at its origin from the external iliac vessels. The composite allograft was then transplanted into a second recipient cadaver abdomen, where segmental intercostal thoracolumbar nerves were similarly prepared using the multilayered component separation technique (see Fig 15). Simulated osteosynthesis between donor and recipient ribs was carried out using Stryker fixation plate and screws (Stryker, 2825 Airview Boulevard, Kalamazoo, Michigan) (see Fig 16).

## RESULTS

The average size of the hexagonal abdominal wall graft harvested was 845 ± 205 cm^2^. The length of abdominal wall procurement was 110 minutes. As the skin flaps were elevated laterally to medially, at least 2 latex-filled perforators from the underlying deep inferior epigastric pedicle were easily identified on both cadaver hemiabdomens.

The 2-layered component separation technique readily allowed for the rapid isolation of the segmental intercostal thoracolumbar nerves. On 1 cadaver, 4 nerves were easily dissected on each side while the other cadaver yielded 3 nerves. The average length of the skeletonized thoracolumbar nerves was 5.7 ± 1.2 cm. We expect that with continued careful dissection each spinal level's intercostal contribution could be identified and that longer lengths of donor nerve could be obtained.

When completing the graft harvest, 6-cm segments of the 3 inferior most vertebrochondral ribs were removed with the rectus muscle attached using a Stryker reciprocating power saw and osteotome. The deep inferior epigastric vessels were transected at their attachment to the external iliac vessels at a length of 4.40 ± 0.10 cm. Upon flap transfer, nerve coaptation was easily simulated between donor and recipient intercostal levels, and that osteosynthesis with internal fixation provided for stable bone to bone contact.

## DISCUSSION

If tissue coverage itself, without motor function, is the end point for a large abdominal wall composite defect such as a hernia, then there are an abundance of techniques already described (ie, pedicled anterolateral thigh flap, tensor fascia lata flaps, bridging biologic mesh, and even skin grafting to bowel). However, if the goal is restoration of a functional abdominal wall with a durable result, then an AW-VCA with motor function is theoretically a better option in the end-stage hernia patient who has already failed multiple previous attempts at reconstruction. A functional graft in essence means a neurotized and vascularized composite graft with the myofascial component being under proper physiologic tension. Such a functional graft would enable the abdominal wall to contribute to respiration, coughing, micturition, flexion movement, and core stability while at the same time preventing herniation of abdominal contents.

Current abdominal wall transplantations serve only as a denervated, poorly functional mechanical retainer of the abdominal contents.^3^^-^6 In fact, using histologic examination and biomechanical testing, Jin et al^16^ demonstrated that the abdominal wall musculature from a denervated allotransplantation atrophied, fibrosed, and had reduced tensile strength within 28 days of transplant in a porcine model. This cadaveric study proposes using a “multilayered” component separation technique as a means to properly identify segmental intercostal thoracolumbar nerves needed to offer a potentially functional and vascularized AW-VCA.

In a cadaveric study, Ramirez et al^17^ described the technique of anterior component separation to primarily close large abdominal wall defects by exploiting the increased mobility of the rectus-internal oblique muscle complex when separated from the external oblique. This technique allows for the midline advancement of the rectus abdominis musculature with minimal donor site morbidity. Our “multilayered” component separation for AW-VCA uses the same principles but varies from the standard technique and serves a different purpose; to carefully and properly identify the nerves in a deeper layer of the abdominal wall.

The first difference between techniques is that component separation, when used to repair abdominal wall defects with native tissue, is started by elevating broad skin flaps through midline laparotomy, progressing in a medial to lateral direction to gain access to the lateral abdominal wall. Conversely, the goal in allotransplantation is to preserve the midline structures and overlying blood supply to the skin. Thus, skin flaps are actually based medially on the deep inferior epigastric perforators and elevated off the underlying abdominal wall from a lateral to medial direction instead. Once the semilunar line is encountered, care is taken to preserve the forthcoming perforators, as they will provide the superficial blood supply to the skin (Huger Zone 1).^18^

Second, instead of just preforming a single layer of separation, allowing access into the avascular plane between the external and internal oblique muscles, a “multilayered” separation is necessary. Continued separation of the internal oblique and transversus abdominis muscle exposes the thoracolumbar neurovascular bundles. The thoracolumbar nerves, T7 to L4, provide the motor innervation to the abdomen necessary for a functional graft. The separation for the second layer was initiated at a point 2-cm lateral to the semilunar line by carefully dissecting through the internal oblique musculature until the plane between the 2 muscles was identified. This location was chosen to avoid damaging the thoracolumbar nerves as they penetrate through the semilunar line.

In our cadaveric study, we demonstrated that it is feasible to identify and to skeletonize adequate length of the thoracolumbar nerves. Through this novel approach, the thoracolumbar nerves could be easily isolated, harvested, and used for coaptation with the similarly prepared recipient thoracolumbar nerves. The longer the segment of nerve that can be harvested from a donor, the easier it will be to perform a neurorrhaphy with the corresponding recipient nerve, especially if the donor has considerable abdominal domain loss. Successful neurotization and implantation of an innervated graft would yield the possibility of a functional abdominal wall.

Another novel concept proposed by our cadaveric study is incorporating bone as part of the allograft. Immunosuppression regimens used in the abdominal wall combined with visceral or solid organ transplants result in an acceptably low rate of graft rejection for both components of the allograft, but the use of these regimens in an otherwise healthy individual with end-stage hernia poses significant ethical considerations. The potential advantages of including vascularized bone marrow are providing a more stable fixation of the allograft, setting the graft under physiologic tension, and reducing immunosuppressive requirements by inducing chimerism.^14^^,^^15^ We found that harvesting only 1 rib with the graft yielded insufficient insertion of the rectus abdominis muscle. Therefore, 3 ribs were harvested with the graft to include a broader insertion of the rectus muscle and allow for a stable fixation under physiologic tension. The ribs though may provide insufficient bone marrow for any chimeric results. In future studies, we will consider the idea of chimeric flap design to include the iliac crest as a bone marrow source. There is, however, potential harm in including ribs in the allograft because operative times would increase. There would also be an increased risk for ischemic complications at the superior portion of the graft as it would be furthest from the inferiorly based blood supply.

An alternative strategy for reducing the antigenicity of the flap itself would be to limit the amount of skin transplanted. Skin presents the highest concentration of antigens, naturally lending itself to higher rates of rejection. We suggest that de-epithelializing the graft could decrease the immunosuppressive requirements necessary. The wound could be regrafted with epidermal autografts or split-thickness skin autograft. In addition, we further imagine that an entirely skinless flap could be harvested from a donor, further decreasing the immunosuppressive requirements. Such a recipient patient could be covered with tissue expanded flaps, tissue rearrangement using thigh flaps, or simply with skin advancement flaps in the recipient. Cosmetically, this concept may be appealing as it provides for local tissue match with respect to surrounding skin quality, color, and tone.

To address the possibility of insufficient vascular supply within the distal graft, the graft could be supercharged with a superior vascular anastomosis based on the lowermost subcostal/intercostal vessels, the superior epigastric system, and/or the superficial inferior epigastric system in addition to the primary vascular pedicle from the deep inferior epigastric vessels bilaterally. It is important to include the deep inferior epigastric system in any graft combination because it is the dominant blood supply to the rectus abdominis muscle. Even though the rectus abdominis muscle can survive with only the superior epigastric system, the perforators that supply the overlying skin, specifically the Huger zone 1 perforators, are primarily supplied by the deep inferior epigastric system.^18^ Identification and evaluation of the vascular territories and overall flap perfusion intraoperatively during the harvest and implantation will be essential to the success of the proposed AW-VCA. Laser-assisted near infrared indocyanine green angiography will be an essential tool for the transplantation of this proposed flap from theory into practice.^19^

It should be noted that incisional hernias are one of the most common types of hernias experienced by patients. It would prove counterproductive and be seen as a failure if a patient's hernia was repaired with an AW-VCA only to have them return with incisional hernia complications, especially after possibly exposing them to an immunosuppressive regiment. However, we argue that having an incisional hernia is more manageable than having total loss of the abdominal domain. Our group has shown that using biological mesh as a primary reinforcement of suture lines reduces the incidence of incisional hernias and also reduces postoperative wound infection rates in immunosuppressed posttransplant patients.^20^ Hence, it may be necessary to reinforce the AW-VCA with a biological mesh.

Expanding the indications for AW-VCA to include the end-stage hernia patient is technically possible. Similar to the challenges previously faced with lower extremity VCAs, much work remains to be done to refine the AW-VCA technique and identify the most appropriate patients for this procedure.^21^^,^^22^ First, we have only illustrated that it is anatomically feasible to harvest an innervated AW-VCA. Animal studies are needed to describe the functionality of such an innervated AW-VCA. Definition and analysis of the psychosocial impact of end-stage hernia disease through a quality-of-life evaluation is another essential step in the process. The study would assess the patients’ desire to undergo such a radical treatment, especially if immunosuppressive requirements cannot be minimized. Our multiple recurrent hernia patients have notable biophysical dysfunction and psychosocial morbidity as a result of their disease, and we suspect there would be a large number of these patients willing to accept immunosuppression for improved quality of life. The key to that improved quality of life is to provide both form and function for these patients, and our hope is that this cadaveric study demonstrates anatomic feasibility for just such a goal.

## CONCLUSIONS

Vascularized composite allotransplantation is an established and rapidly expanding field that promotes the idea of quality of life in patients with severe pathology such as recalcitrant facial deformity and severe hand dysfunction. While abdominal wall transplantation is already a reality, we believe the provision of functional innervation may expand the indication to those patients suffering from debilitating end-stage hernia disease.

Demonstrated here is a novel approach to the abdominal wall—using principles of component separation technique—to easily dissect the crucial segmental intercostal innervation within the deep lateral abdominal wall. This technique is feasible in a cadaver model indicating the possibility of osteomyofasciocutaneous free flap for allotransplantation. Furthermore, we illustrate the ability to include bone with orthopedic osteosynthesis for the purpose of inducing marrow-derived chimeric tolerance.
